# Torpedo Retinopathy

**DOI:** 10.18502/jovr.v15i2.6736

**Published:** 2020-04-06

**Authors:** Ramesh Venkatesh, Kushagra Jain, Arpitha Pereira, Thirumalesh, Naresh Kumar Yadav

**Affiliations:** Department of Retina and Vitreous, Narayana Nethralaya, Bangalore, India

**Keywords:** Imaging, Maculopathy, Retinopathy, Torpedo

## Abstract

**Purpose:**

Torpedo lesions in the retina are rare. This study aimed to investigate torpedo-shaped lesions in the retina in an adult population and to determine the spectrum and features of the disease.

**Methods:**

The review of a database for clinical diagnosis identified nine patients who were diagnosed with torpedo-shaped lesions in the retina between June 2017 and February 2019. Fundus photography and optical coherence tomography (OCT) imaging were used to analyze the cases. Multicolor imaging was also performed.

**Results:**

Nine patients with torpedo-shaped lesions in the fundus were identified. Fundus images revealed that the lesion involved the macula in six eyes; in the remaining three eyes, the lesion was present outside the macula. OCT identified six patients with type 1 torpedo lesions, one with type 2, and two with type 3. On multicolor imaging, the lesion was visualized as a region of increased reflectance in blue, green, and infrared light in all eyes, with notably increased infrared reflectance in eyes with focal choroidal excavation. Choroidal neovascular membrane was evident in one patient on OCT angiography.

**Conclusion:**

Torpedo lesions in the retina can occur away from the macula and exhibit features similar to those of torpedo maculopathy. As such, the authors propose a change in the nomenclature for torpedo lesions in the retina from “torpedo maculopathy” to “torpedo retinopathy.”

##  INTRODUCTION

Congenital pathologies of the retinal pigment epithelium (RPE) include torpedo maculopathy (TM), congenital hypertrophy of the RPE (CHRPE), and RPE lesions apparent in Gardner syndrome associated with familial adenomatosis polyposis and colon polyps. Each of these pathologies has classical clinical descriptions, separating one from the other on the basis of the lesion characteristics.^[1]^ In TM, as described by Rossman and Gass in 1992, the classical clinical description includes a non-progressive, unilateral, horizontally oval, hypopigmented lesion(s) temporal to the fovea, with the tip pointing toward the central macula.^[2,3]^ CHRPE is clinically described as an asymptomatic, flat, variably pigmented lesion at the level of the RPE and may have depigmented lacunae and/or a surrounding hypopigmented halo. The lesions are usually solitary; however, very rarely, they may occur in clusters sometimes referred to as congenital grouped pigmentation of the RPE or “bear tracks.”^[4]^ CHRPE has no systemic associations and rarely occurs in the macula (1%) but may enlarge over time. The RPE lesion in systemic conditions, such as Gardner syndrome, are usually small ( < 1 mm), multiple, and bilateral, and often located in the midperiphery.^[5]^ However, in the clinic, there are a group of cases in which the lesions do not necessarily meet the diagnostic criteria of any of these three clinical entities. The aim of the present case series was to illustrate the spectrum of torpedo-shaped lesions in the retina, patient demographics, coinciding systemic conditions, and variable imaging appearances in patients in a tertiary eye hospital in South India and to explain the possible etiopathogenesis associated with these lesions.

##  METHODS 

The analysis of medical records at a tertiary eye care hospital in South India identified nine patients with torpedo-shaped lesions in the retina between June 2017 and February 2019. Torpedo lesions were characterized as oval, hypopigmented, or hyperpigmented lesions at the level of the RPE, with the tip of the lesion usually directed toward the macula. The medical records of these patients were reviewed and demographic information, clinical presentation, and medical history were recorded. All patients underwent fundus imaging using the Topcon TRC-50DX (Topcon Corporation, Tokyo, Japan) or Optos (Daytona, Optos PLC, United Kingdom) devices. All patients underwent optical coherence tomography (OCT), blue wavelength fundus autofluorescence (FAF), and multicolor (MC) imaging using a confocal scanning laser ophthalmoscope and a Heidelberg Spectralis HRA-OCT (Heidelberg Engineering, Dossenheim, Germany). Fluorescein angiography (FA) (Spectralis, Heidelberg Engineering, Heidelberg, Germany) and OCT angiography using the RTVue-XR Avanti (Optovue, Fremont, CA, USA) device were performed in one patient while adaptive optics (AO) imaging using the Rtx1 flood illumination-based fundus camera (Imagine Eyes, Orsay, France) was performed in two patients. Approval was obtained from the Narayana Nethralaya Research and Ethics Committee. Written informed consent was obtained from all patients to include anonymized data in the present research.

##  RESULTS

In the present series, nine eyes diagnosed with torpedo-shaped lesions were included. The average age of the patients (four males, five females) was 45.3 years (range, 20–67 years). All cases were unilateral, with five presenting lesions in the right eye and four with lesions in the left eye. All patients were asymptomatic on presentation.

Visual acuity in the affected eye ranged from 6/5 to 6/12 on the Snellen visual acuity chart. The eye with 6/12 vision had significant cataract. None of the patients had any associated systemic conditions.

On color fundus photography, all lesions were hypopigmented to varying degrees and were located in the temporal retina. They were oval and elongated in the horizontal axis. All lesions had a nasal pointed tip directed toward the posterior pole and a temporal rounded margin. There were varying degrees of associated hyperpigmented RPE changes surrounding the lesions. The exact location of the lesion was immediately adjacent to the fovea in four eyes, adjacent to the macula but away from the fovea in two eyes, immediately within the retinal arcade in one eye, and outside the retinal arcade in two eyes.

**Figure 1:**
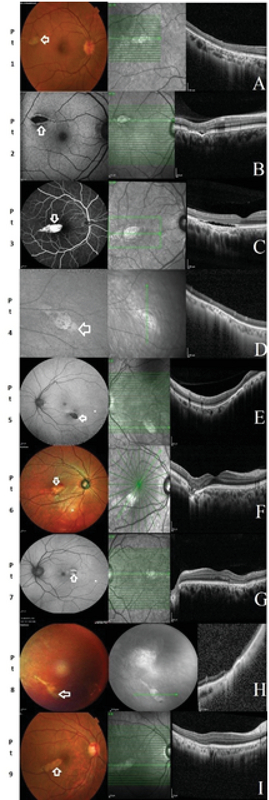
Fundus and OCT imaging of torpedo lesions (patients 1–9). The torpedo lesion in each panel is marked on the fundus image with white arrow. In panels, C, F, G and I, the torpedo lesion is located adjacent to the fovea. In panels B and E, the lesion is located adjacent to the macula but away from the fovea. In panel A, the lesion is just within the retinal arcade, and in panels D and H, the lesion is outside the retinal arcade. In every case, the OCT scan passing through the torpedo lesion is depicted.

OCT was performed in all patients. The following features on OCT through the torpedo lesion were searched for in each case: thinning of the inner retina; thinning of the outer nuclear layer; thinning of the inter-digitation and ellipsoid zones; presence of subretinal cleft or cavitation; and presence of hyper-reflectivity of choroid, choroidal thinning, focal choroidal excavation, and choroidal neovascular (CNV) membrane. On analysis, it was noted that the inner retina was normal in all eyes except one (patient 8). Thinning of the outer nuclear layer, interdigitation zone, and ellipsoid zone was noted in all cases. Subretinal cavitation/cleft was noted in two cases (patients 2 and 3). The choroid appeared normal on OCT in two eyes (patients 1 and 7). Thinning of the underlying choroid and increased hyper-reflectivity was noted in six cases (except patients 1, 2, and 7). Focal excavation of the choroid was observed in two eyes (patients 2 and 6) [Figure 1].

FAF imaging using the short wavelength blue light identified the lesion as exhibiting hypo-autofluorescence (AF) with hyper-AF boundaries in four eyes (patients 1, 2, 3, and 5), hyper AF in three eyes (patients 4, 7, and 8), and a mixture of hyper- and hypo-AF in two eyes (patients 6 and 9). In three eyes with torpedo lesions, near infrared FAF was also performed, which revealed the lesion to be hyper-AF.

**Figure 2:**
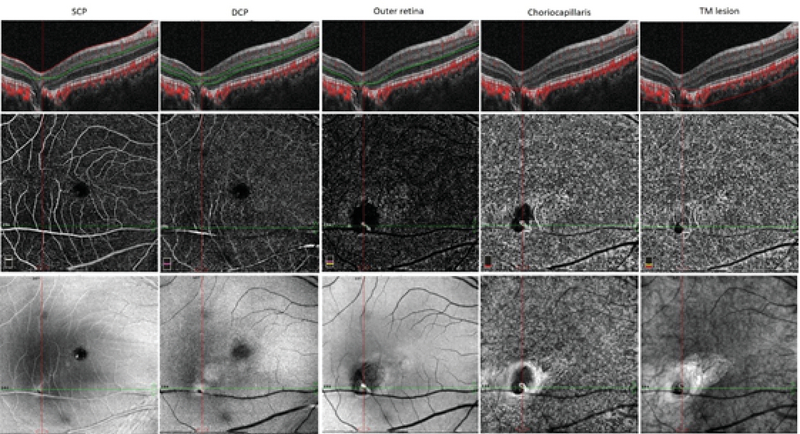
OCT angiography findings of patient 6 showing the torpedo lesion with focal choroidal excavation with presence of choroidal neovascular membrane. Columns 1–5 show the automated segmentation scans through the superficial capillary plexus, deep capillary plexus, outer retina, choriocapillaris, and manual segmentation across the torpedo lesion with focal choroidal excavation, respectively. Note the decreased flow areas and vessel density in the area of torpedo lesion. A choroidal neovascular membrane can be identified as well.

**Figure 3:**
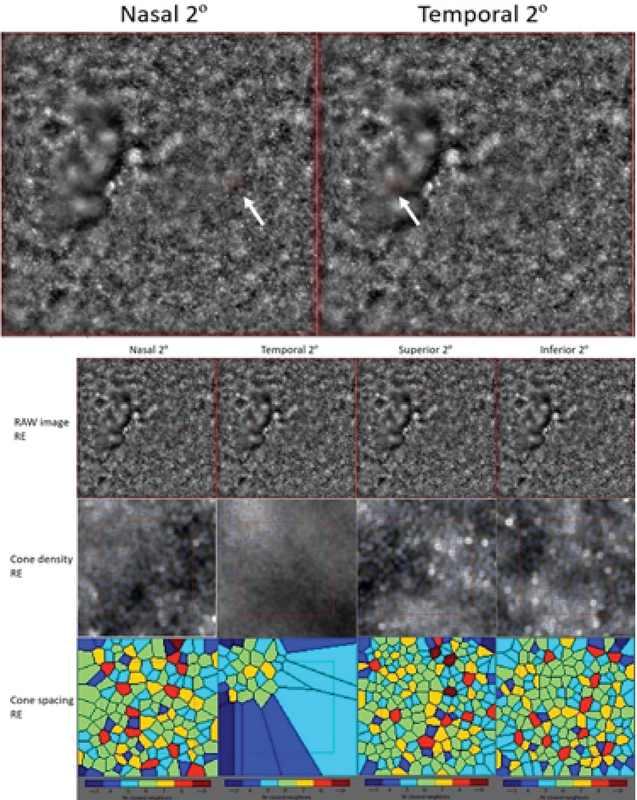
Adaptive optics imaging of the right eye in patient 3 with torpedo lesion. Raw images on adaptive optics imaging showing the exact site of cone density and spacing analysis (bold white arrow). Adaptive optics imaging showing raw images, cone density, and cone spacing of right eye at 2° from the foveal center in all four quadrants.

FA in patient 3 revealed late hyper-fluorescence due to the window defect created by the torpedo lesion. OCT angiography was performed in patient 6 with torpedo lesion and focal choroidal excavation, which revealed a decrease in the vessel density and flow areas in the region of the torpedo lesion, with the presence of a CNV membrane on the choriocapillaris slab [Figure 2]. AO imaging in patients 3 and 9 revealed reduced cone density and increased cone spacing in the region of the lesion [Figure 3].

MC imaging was performed in all eyes using the confocal scanning laser ophthalmoscopy (cSLO) platform (Heidelberg Spectralis HRA-OCT, Heidelberg Engineering, Dossenheim, Germany) with a scanning field of 30° or 55° depending on the location of the lesion. The lesion was observed as a region of increased reflectance in blue, green, and infrared light in all eyes, with notably increased infrared reflectance in eyes with focal choroidal excavation. On MC images, the lesion was observed as an area of bright discoloration in all eyes. Demographic information and imaging features are summarized in Table 1.

##  DISCUSSION 

In the current case series, there appeared to be similarities in terms of clinical and imaging features of torpedo lesions. All cases exhibited a hypopigmented lesion in the temporal retina, with some located immediately adjacent to the fovea and some lying outside the retinal arcade. In all cases, the lesion was oval, with a characteristic pointed tip aimed toward the macula. Thus, our case series demonstrated two varieties of torpedo lesions: those involving the macula (routinely known as TM) and those lying outside the macula. The differentials for torpedo lesions lying outside the retinal arcade were CHRPE and RPE lesions, observed in systemic disorders such as Gardner syndrome and

**Table 1:** Demographic, clinical, and imaging profile of patients with torpedo lesions in retina


**Patient**	**Age/Sex**	**Affected eye**	**Visual acuity**	**Color fundus**	**Fundus AF**	**OCT**	**FFA**	**Multicolor imaging**	**Adaptive optics imaging**
1	43/F	RE	6-Jun	• Just inside superotemporal arcade	Hypo-AF with hyper borders	• Thinning of the outer nuclear layer	Not done	Hyper reflectance in BR, GR, IR	Not done
		• Hypopigmented	• Thinning of inter digitation zone		
			• Thinning of ellipsoid zone		
2	54/M	RE	6-Jun	• Temporal to macula	Hypo-AF with hyper borders	• Thinning of the outer nuclear layer	Not done	Hyper reflectance in IR	Not done
		• Hypopigmented	• Thinning of inter digitation zone		
			• Thinning of ellipsoid zone		
			• Presence of subretinal cleft/cavitation		
			• Focal choroidal excavation		
3	38/M	RE	6-Jun	• Temporal to fovea	Hypo-AF with hyper borders	• Thinning of the outer nuclear layer	Late stage hyperfluoroscence	Hyper reflectance in BR, GR, IR	Done
		• Hypopigmented	• Thinning of inter digitation zone		
			• Thinning of ellipsoid zone		
			• Presence of subretinal cleft/cavitation		
			• Thinning of inner choroid		
4	65/F	LE	9-Jun	• Outside the infero temporal arcade	Hyper-AF	• Thinning of the outer nuclear layer	Not done	Hyper reflectance in BR, GR, IR	Not done
		• Hypopigmented	• Thinning of inter digitation zone		
			• Thinning of ellipsoid zone		
			• Thinning of inner choroid		
5	67/M	LE	6-Jun	• Inferotemporal to macula	Hypo-AF with hyper borders	• Thinning of the outer nuclear layer	Not done	Hyper reflectance in BR, GR, IR	Not done
		• Hypopigmented	• Thinning of inter digitation zone		
			• Thinning of ellipsoid zone		
			• Thinning of inner choroid		
6	20/F	RE	6-Jun	• Inferotemporal to fovea	Mixture of hypo- and hyper-AF	• Thinning of the outer nuclear layer	Not done	Hyper reflectance in IR	Not done
		• Mix of hypo- and hyperpigmentation	• Thinning of inter digitation zone		
			• Thinning of ellipsoid zone		
			• Thinning of inner choroid, focal choroidal excavation		
7	33/F	LE	6-Jun	• Temporal to fovea	Hyper-AF with focal hypo-AF	• Thinning of the outer nuclear layer	Not done	Hyper reflectance in BR, GR, IR	Not done
		• Hypopigmented	• Thinning of inter digitation zone		
			• Thinning of ellipsoid zone		
8	63/F	LE	12-Jun	• Outside the infero temporal arcade	Hyper-AF	• Thinning of inner retina	Not done	Hyper reflectance in BR, GR, IR	Not done
		• Hypopigmented	• Thinning of the outer nuclear layer		
			• Thinning of inter digitation zone		
			• Thinning of ellipsoid zone		
			• Thinning of inner choroid		
9	25/M	RE	6-Jun	• Temporal to fovea	Mixture of hypo- and hyper-AF	• Thinning of the outer nuclear layer	Not done	Hyper reflectance in BR, GR, IR	Done
		• Hypopigmented	• Thinning of inter digitation zone		
			• Thinning of ellipsoid zone		
			• Thinning of inner choroid		

M, male; F, female; RE, right eye; LE, left eye; AF, auto fluorescence; OCT, optical coherence tomography; FFA, fundus fluorescein angiography; BR, blue reflectance; GR, green reflectance; IR, infrared reflectance									

familial adenomatous polyposis. CHRPE is a flat congenital RPE lesion that appears pigmented or nonpigmented and characteristically has rounded or scalloped margins.^[6]^ Solitary CHRPE is located most often in the equatorial or peripheral fundus, randomly in various quadrants, and rarely in the macula (1%).^[1,6]^ The RPE abnormalities associated with familial adenomatous polyposis and Gardner syndrome are also similar to TM. However, individuals with familial adenomatous polyposis manifest a random distribution in the fundus and are often bilateral, significantly smaller, and more irregular in shape.^[1,7]^ The clinical description of torpedo lesion in our cases did not match with either CHRPE or RPE lesions associated with systemic disorders.

Although a torpedo lesion is classically described as a solitary oval lesion with variable pigmentation, satellite lesions can also occur, as observed in patients 1 and 5 of our series.

All satellite lesions reported to date have been located temporally and are usually small, round, and flat. OCT imaging through the satellite lesions reveals changes similar to those in the main torpedo lesion. Satellite lesions may add to our understanding of the morphology of these lesions because they appear to be an extension of the main lesion.

The common features described on OCT in eyes for scans passing through the TM lesion included thinning of the outer nuclear layer, interdigitation zone, ellipsoid zone, and inner choroid in all cases. Additional features described by Wong et al,^[8]^ such as the presence of subretinal cavitation, have helped in classifying torpedo lesions into types 1 and 2. Focal retinal and choroidal excavation in torpedo lesions were described as type 3 OCT findings by Tripathy et al.^[9]^ Similar OCT features were noted for lesions lying outside the macula in the present series. No subretinal cavitation/cleft or focal excavation of the retina and/or choroid was noted in eyes in which the torpedo lesions were present outside the macula. MC imaging signatures of torpedo lesions in the series coincided with data previously published by our group.^[10]^

The etiology of torpedo-shaped lesions in the retina remains speculative. Due to the consistent location of torpedo lesions in previous reports of TM, the most commonly accepted hypothesis is a persistent defect in the development of RPE in the fetal temporal bulge.^[11]^ In one patient who underwent AO imaging, we found fewer cone photoreceptors and increased cone spacing in the region of the torpedo lesion. However, the presence of lesions in regions other than the macula in our series explains the need to determine an alternative hypothesis for the development of torpedo lesions. The most probable explanation for the presence of these lesions could be abnormal choroidal vasculature, as observed on OCT angiography as a loss of choriocapillaris in the area of the lesion.^[12,13]^ A similar finding was also noted in our case. However, in this series, OCT angiography scans through the lesions located outside the macula could not confirm the probable choroidal vascular hypothesis.

Although, patients with torpedo lesions are asymptomatic and experience a benign and non-progressive disease course, the CNV membrane in TM has been described.^[14,15]^ The presence of CNV can lead to disturbance in vision and deterioration in visual acuity. Patient 6 in our series developed CNV. Previous studies have suggested that patients exhibiting outer retinal damage and choroidal thinning have a higher risk for developing CNV. Thus, we suggest that CNV development in torpedo lesions can occur in patients with type 2 or 3 findings on OCT.

To conclude, we present a series of cases in an adult population with torpedo-shaped lesions in the retina. Contrary to previous reports in which torpedo lesions were classically described at the macula, in this series, we present cases located in the temporal half of the retina, away from the macula, outside the arcades, and exhibiting phenotypic and imaging features similar to those of TM. Based on the findings from the current case series, we believe that torpedo lesions in the retina occur secondary to changes in the choroidal vasculature. Furthermore, we propose a change in the nomenclature of torpedo lesions in the retina from “torpedo maculopathy” to “torpedo retinopathy.”

##  Financial Support and Sponsorship

Nil.

##  Conflicts of Interest

There are no conflicts of interest.
